# A Novel De Novo *MTM1* Insertion Frameshift Variant Causes X‐Linked Myotubular Myopathy in a Chinese Female

**DOI:** 10.1002/mgg3.70318

**Published:** 2026-10-02

**Authors:** Lijun Chen, Yingxiao Bao, Gonglu Liu

**Affiliations:** ^1^ Department of Medical Genetics and Center for Rare Diseases The Second Affiliated Hospital, Zhejiang University School of Medicine Hangzhou Zhejiang China; ^2^ Department of Nursing The Second Affiliated Hospital, Zhejiang University School of Medicine Hangzhou Zhejiang China

**Keywords:** de novo variant, *MTM1*, respiratory insufficiency, symptomatic female carrier, X‐linked myotubular myopathy

## Abstract

**Background:**

*MTM1*‐related X‐linked myotubular myopathy (XLMTM) typically affects males; however, symptomatic female carriers may occur.

**Methods:**

We evaluated a 24‐year‐old woman with infantile hypotonia followed by slowly progressive, asymmetric proximal weakness with facial and ocular involvement. Clinical assessment, pulmonary function testing, electromyography, lower‐limb muscle MRI, and muscle biopsy were performed. Next‐generation sequencing and parental segregation analysis were conducted.

**Results:**

The patient developed progressive functional decline leading to wheelchair dependence. Investigations showed moderate‐to‐severe restrictive ventilatory impairment, a myopathic pattern on electromyography, and extensive fatty replacement on muscle MRI. Muscle biopsy demonstrated histopathological features consistent with centronuclear myopathy. Genetic testing identified a heterozygous *MTM1* insertion, c.1266_1267insATGGTGATAAAAACCATTCA, predicted to cause a frameshift (p.Gly423MetfsTer48). The variant was absent in both parents, supporting a de novo event. To our knowledge, this *MTM1* variant has not been previously reported.

**Conclusion:**

This case expands the *MTM1* variant spectrum and highlights that de novo *MTM1* frameshift variants can underlie clinically significant XLMTM manifestations in females, underscoring the value of genetic testing in symptomatic women with centronuclear myopathy features.

## Graphical abstract

A 24‐year‐old heterozygous woman presented with lifelong hypotonia and slowly progressive, asymmetric axial and limb‐girdle weakness with facial/ocular involvement and restrictive ventilatory impairment (FVC 53.12% predicted). Multimodal evaluation (EMG, muscle MRI, and whole‐exome sequencing [WES]) identified a de novo MTM1 frameshift variant (NM_000252.3:c.1266_1267insATGGTGATAAAAACCATTCA; p.(Gly423MetfsTer48)), confirmed by Sanger sequencing and absent in parental/sibling blood DNA. This case broadens the phenotype of symptomatic female MTM1 carriers and supports proactive respiratory surveillance.

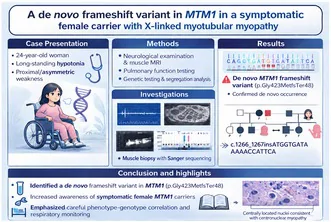

## 1 Introduction

X‐linked myotubular myopathy (XLMTM; OMIM #310400) is a congenital myopathy caused by pathogenic variants in the *MTM1* gene, encoding myotubularin, a phosphoinositide phosphatase critical for muscle fiber maintenance and triad organization (Al‐Qusairi et al. 2009; Laporte et al. 1996; Lawlor and Dowling 2021). The disorder is classically recognized in males with severe neonatal hypotonia and early respiratory insufficiency, yet accumulating genotype–phenotype data support a broader *MTM1*‐related disease spectrum beyond the canonical neonatal‐lethal presentation (Annoussamy et al. 2019; Lawlor and Dowling 2021).

Historically, heterozygous females were often labeled “asymptomatic carriers”, however, multiple cohorts and deep‐phenotyping studies demonstrate that a meaningful subset of females develop myopathy ranging from mild limb‐girdle weakness to profound disability (Biancalana et al. 2017; Souza et al. 2020). In symptomatic females, recurring diagnostic clues include asymmetric muscle weakness and/or skeletal size, facial weakness, ptosis/ophthalmoparesis, and slowly progressive functional decline (Biancalana et al. 2017; Souza et al. 2020). Muscle pathology may show centronuclear myopathy features such as centralized nuclei and the “necklace fiber” pattern—an important histologic clue that can direct genetic analysis toward *MTM1* in appropriate clinical contexts (Bevilacqua et al. 2009; Biancalana et al. 2017). Phenotypic expression in females is thought to be influenced by tissue‐specific factors including skewed X‐chromosome inactivation and mosaicism, although the relationship between X‐inactivation measured in blood and clinical severity can be variable (Biancalana et al. 2017; Souza et al. 2020).

Respiratory involvement, a major driver of morbidity in congenital myopathies, is often underrecognized in slowly progressive cases, especially among female patients with X‐linked myopathies. Contemporary consensus guidance recommends serial pulmonary function testing (e.g., FVC/SVC and respiratory pressures) and timely initiation of noninvasive ventilation when indicated (Wang et al. 2012). Parallel to advances in natural history characterization and outcome measures (including muscle MRI and functional scales) in *MTM1*‐related disease, translational efforts—most notably gene replacement strategies—have further intensified interest in accurate phenotyping and longitudinal monitoring, while also highlighting safety considerations in this population (Annoussamy et al. 2019; Shieh et al. 2023).

Here, we report a young woman with long‐standing progressive myopathy characterized by craniofacial/ocular involvement, asymmetric weakness, and clinically significant restrictive ventilatory impairment, in whom a heterozygous *MTM1* insertion variant was identified on genetic testing with family segregation analysis.

## 2 Materials and Methods

### 2.1 Subjects

A 24‐year‐old female patient was recruited from the Second Affiliated Hospital of Zhejiang University School of Medicine in 2024. The proband underwent a series of investigations, including biochemical tests, magnetic resonance imaging (MRI), electromyography (EMG), muscle biopsy for histopathological analysis, and next‐generation whole‐exome sequencing. This study was approved by the Ethics Committee of Second Affiliated Hospital, Zhejiang University School of Medicine and the informed consent was signed by the participant.

### 2.2 Genetic Analyses

Genomic DNA was extracted from peripheral blood by using a DNA isolation kit (Qiagen, Germany). Whole‐exome sequencing (WES)was performed using the Agilent SureS‐ elect Human All Exome V6 kit (Agilent Technologies Inc., Canada) on an Illumina HiSeq X Analyzer (Illumina, USA) with 150‐bp paired‐end reads. Sanger sequencing and co‐segregation analysis were further carried out in the proband and all available family members. Variant pathogenicity analysis was based on the ACMG standards and guidelines (Richards et al. 2015).

## 3 Results

### 3.1 Clinical Phenotype and Neurological Examination

The patient exhibited generalized neonatal hypotonia identified by her family, alongside poor head control and torticollis. She attained independent walking at age 4 yet remained prone to unsteady gait and recurrent falls. Toe‐walking emerged at age 7 and gradually progressed to bilateral talipes equinovarus, accompanied by concomitant symptoms including impaired overhead arm elevation, incomplete eyelid closure, masticatory weakness, and facial asymmetry characterized by a hypoplastic right side compared with the left. Her motor function steadily deteriorated during adolescence: she required assistance for ambulation between ages 15 and 18 and became wheelchair‐bound at 19, at which point she could still stand unaided for roughly 2 mins. She lost the capacity for unsupported standing 1 year before admission. She underwent bilateral corrective orthopedic surgery for equinovarus foot deformities, which yielded no substantial improvement in motor function postoperatively.

At admission, neurological examination revealed severe asymmetric weakness mainly involving axial and limb‐girdle muscles, quantified using the Medical Research Council (MRC) scale. Neck flexion was Grade 1, and neck rotation was Grade 3. Upper limb proximal strength was Grade 3 bilaterally, with distal strength graded 3 on the right and 3+ on the left. The patient had a history of bilateral ankle arthrodesis, which prevented evaluation of distal lower limb strength. Assessable proximal lower limb muscles showed bilateral iliopsoas weakness (Grade 1), right quadriceps strength of Grade 4‐, left quadriceps strength of Grade 3‐, and bilateral hamstring strength of Grade 4‐. Prominent extraocular, facial, and bulbar muscle involvement was present, including mild bilateral ocular abduction restriction, incomplete eyelid closure, rightward tongue deviation, a high‐arched palate, and masticatory weakness, while cheek puffing was preserved. Deep tendon reflexes were diminished to absent in all limbs, with negative bilateral Hoffman's and Babinski's signs. The patient also presented with bilaterally small hands and feet and chronic foot deformities. Clinical photographs are shown in Figure 1A.

**FIGURE 1:**
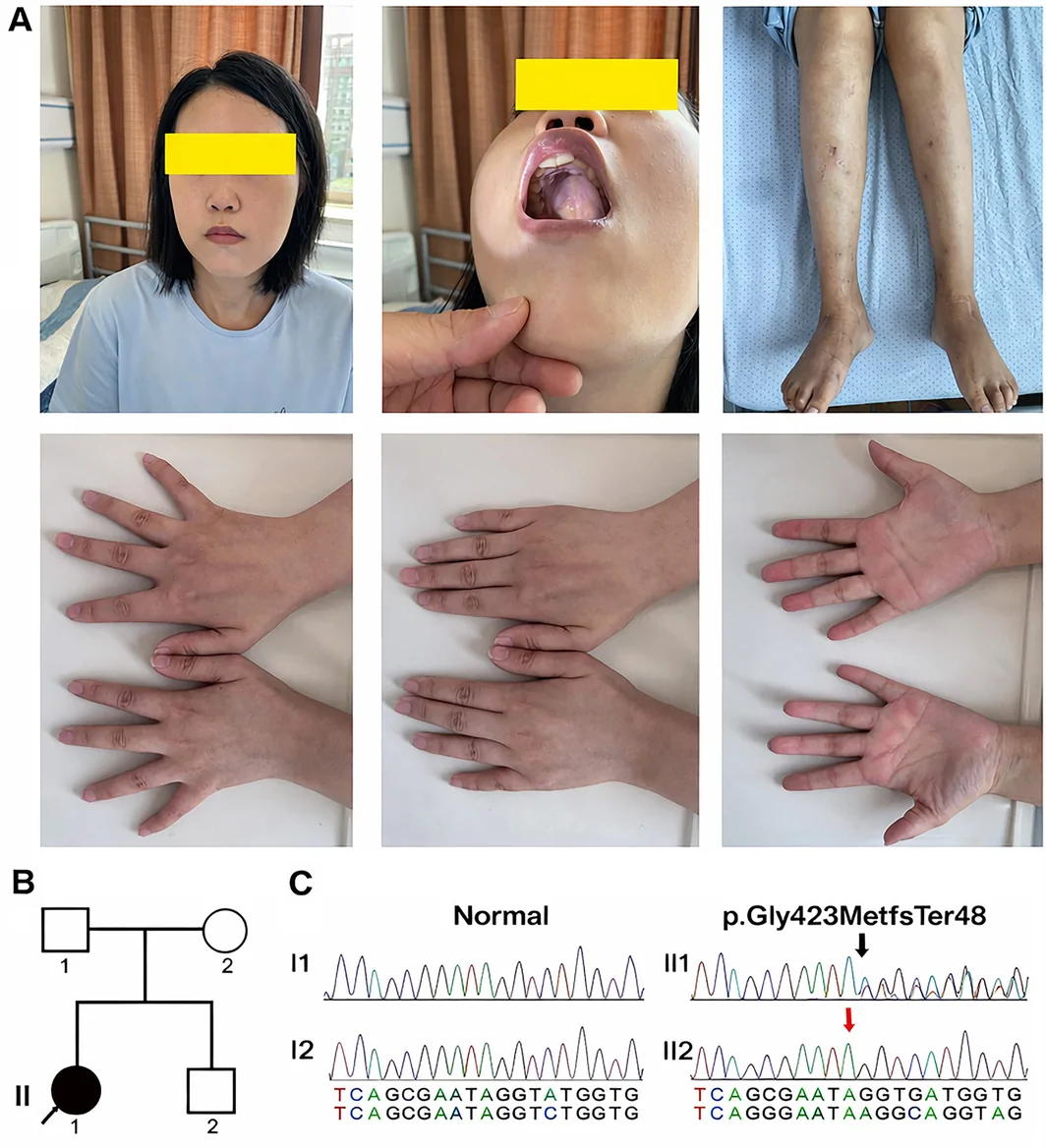
Pedigree and genetic analysis of the proband. (A) Representative clinical photographs showing craniofacial and distal involvement, including facial asymmetry (right face smaller than the left), a high‐arched palate, and small hands/ft with distal muscle atrophy. (B) Pedigree of the family; the proband is indicated by an arrow, and filled symbols denote affected individuals. Squares represent males and circles represent females. (C) Sanger sequencing chromatograms confirming the MTM1 frameshift variant in the proband (arrow) and its absence in both parents, consistent with a de novo event (variant annotation as indicated).

### 3.2 Genetic Findings and Segregation

Genetic testing identified a heterozygous *MTM1* insertion variant, c.1266_1267 insATGGTGATAAAAACCATTCA. The variant is located at chrX:149828142–149828143 based on the transcript reference sequence NM_000252.3, which results in a frameshift mutation p.(Gly423MetfsTer48). No compound heterozygous or homozygous pathogenic variants in other neuromuscular disease‐related genes were identified in this patient. Sanger segregation confirmed the variant in the proband and showed no variant detected in peripheral blood DNA from the father, mother, or younger brother, compatible with a likely de novo event in the proband, while parental germline mosaicism could not be fully excluded without deeper molecular testing (Figure 1B,C). According to the 2015 ACMG (Richards et al. 2015) variant classification guidelines, this variant was classified as Pathogenic with the following supporting evidence: (1) PVS1: the 20‐base‐pair insertion causes a frameshift starting at codon Gly423 and introduces a premature termination codon after 48 aberrant residues, leading to severe truncation of the C‐terminal functional domain of myotubularin protein; (2) PM2: this variant is absent from gnomAD, ClinVar, and HGMD databases, with no population carrier frequency recorded; (3) PS2: segregation analysis confirmed a de novo origin in the affected female proband, with both parents and unaffected younger brother negative for the variant, strongly supporting pathogenicity of this novel truncating variant.

### 3.3 Laboratory and Respiratory Assessments

Serum creatine kinase was within the reference range (CK 69 U/L), and available records indicated normal cardiac biomarkers. Routine laboratory tests including complete blood count, thyroid function panel, antinuclear antibodies, fasting blood glucose, and glycated hemoglobin were all within normal limits. Liver function tests including serum transaminases were all within normal limits, and hepatic ultrasound revealed no remarkable abnormalities. Pulmonary function testing demonstrated moderate‐to‐severe restrictive ventilatory impairment (FVC 53.12% predicted), with reported small airway dysfunction and a moderate reduction in diffusion capacity, supporting clinically significant respiratory muscle involvement.

### 3.4 Electrophysiology and Muscle MRI

Electromyography showed a myopathic pattern. Nerve conduction studies suggested possible axonal sensorimotor peripheral neuropathy, predominantly affecting the lower limbs. Repetitive nerve stimulation showed no decrement. Muscle MRI demonstrated chronic myopathic changes with marked atrophy and extensive fatty replacement. Left upper arm muscles (deltoid, biceps, triceps) presented severe fatty infiltration, while the infraspinatus and subscapularis were relatively spared. Bilateral thighs showed asymmetric involvement with far more severe fatty replacement on the left, and the right vastus lateralis was partially preserved. Multiple thigh muscle groups were extensively replaced by fat, with relative sparing of the rectus femoris, sartorius, adductor longus, and semimembranosus. Calf MRI showed heterogeneous fatty infiltration; the left tibialis anterior and peroneal muscles were nearly completely replaced by fat, whereas the soleus and gastrocnemius were mildly affected. (Figure 2A–F).

**FIGURE 2:**
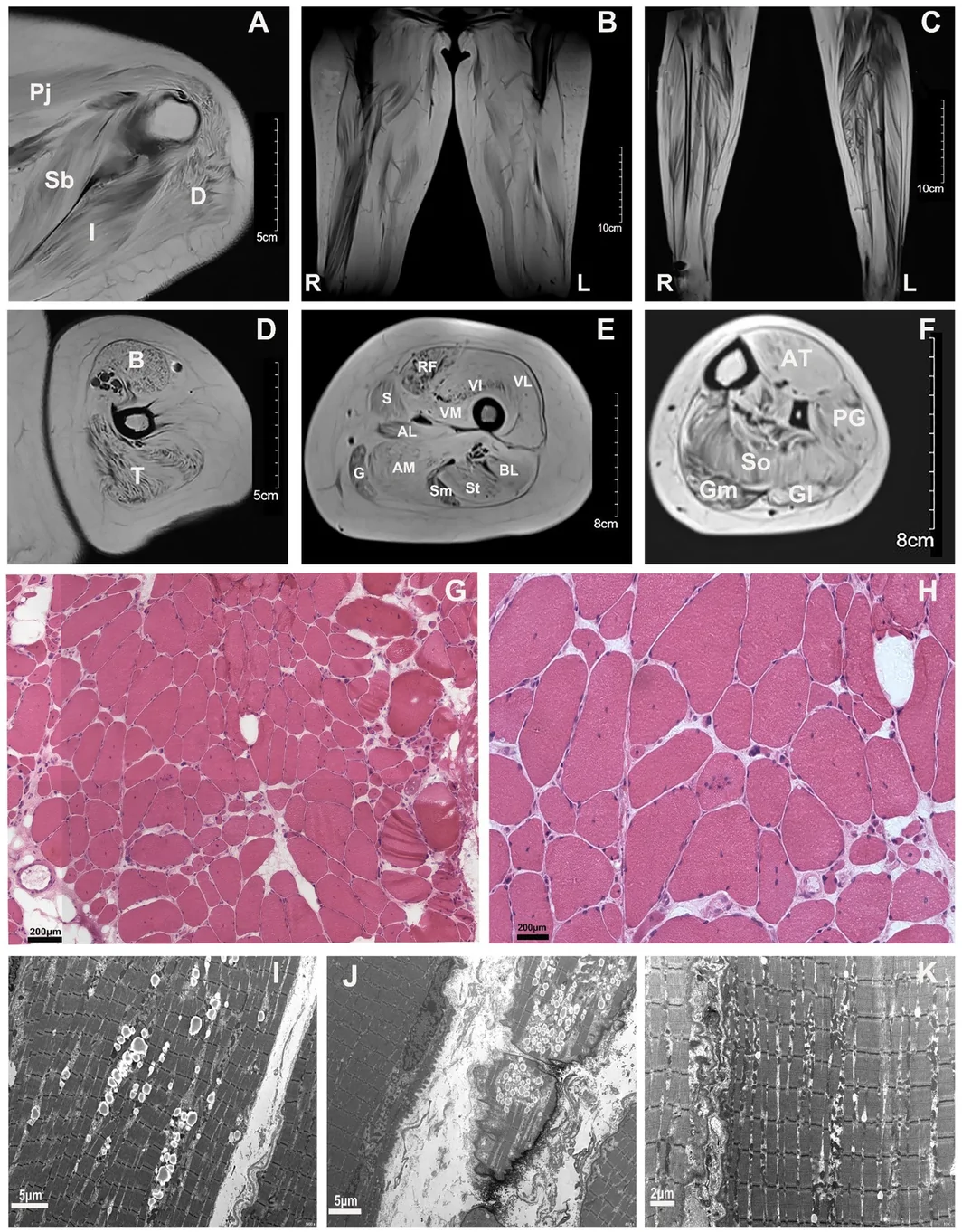
Muscle MRI and histopathological features of the proband. (A, D) MRI of the left upper arm demonstrated muscle atrophy with diffuse fatty infiltration. Partial sparing was noted in the infraspinatus and subscapularis muscles, whereas severe fatty replacement was present within the deltoid, biceps brachii, and triceps brachii. B, Biceps brachii; D, Deltoid; I, Infraspinatus; Pj, Pectoralis major; Sb, Subscapularis; T, Triceps brachii. (B) Coronal MRI of bilateral thighs showed variable degrees of fatty replacement within bilateral lower extremity muscles, with more severe fatty infiltration observed in the left thigh and partial sparing of the right vastus lateralis. (E) Axial MRI of the left lower limb revealed extensive fatty replacement involving the anterior and posterior thigh muscle compartments. Partial sparing was seen in the rectus femoris, sartorius, adductor longus, and semimembranosus muscles. AL, Adductor longus; AM, Adductor magnus; BL, Biceps femoris‐long head; G, Gracilis; RF, rectus femoris; S, Sartorius; Sm, Semimembranosus; St, Semitendinosus; VI, vastus intermedius; VL, vastus lateralis; VM, vastus medialis. (C) Coronal MRI of the calves demonstrated fatty infiltration of varying severity in bilateral crural muscle groups, with relatively mild involvement of the soleus and gastrocnemius muscles. (F) Axial MRI of the left calf showed near‐complete fatty replacement of the tibialis anterior and fibular muscle compartments. The soleus and gastrocnemius also exhibited fatty infiltration of variable grades but were comparatively less severely affected. AT, Tibialis anterior; PG, Peroneal group; So, Soleus; Gm, Gastrocnemius medial head; Gl, Gastrocnemius lateral head. (G, H) Hematoxylin–eosin (HE) stained sections showed marked myofiber size variation, numerous centrally located nuclei and endomysial hyperplasia, without conspicuous necrosis or inflammatory cell infiltration. Compensatory hypertrophic muscle fibers were also identified. (I, J, K) Transmission electron microscopy revealed largely preserved myofibrillar architecture with focal sarcomeric disruption and intermyofibrillar/subsarcolemmal lipid droplet accumulation, without specific inclusions or tubular aggregates.

### 3.5 Histopathological and Ultrastructural Findings

Muscle biopsy showed marked fiber‐size variation with numerous internalized nuclei, but no typical necklace fibers were identified (Figure 2G,H). Electron microscopy demonstrated largely preserved myofibrillar organization with focal sarcomeric disruption, mild myofibrillar splitting/lysis, and occasional atrophic fibers (Figure 2I–K). The sarcolemma showed mild infolding, and myonuclei were increased and clustered. No nemaline rods were identified. A subset of fibers contained increased subsarcolemmal and intermyofibrillar lipid droplets. The interstitium was mildly widened without significant fibrosis, inflammatory infiltration, or capillary basement‐membrane thickening.

## 4 Discussion

XLMTM due to *MTM1* variants is classically a severe male congenital myopathy, but symptomatic females are increasingly recognized (Biancalana et al. 2017; Savarese et al. 2016). We present a symptomatic female harboring a novel, de novo *MTM1* frameshift variant. Our patient showed slowly progressive, asymmetric axial–proximal weakness with craniofacial and ocular involvement, moderate‐to‐severe restrictive ventilatory impairment, a myopathic profile on electrodiagnostic with possible lower‐limb axonal neuropathy, and diffuse fatty replacement on muscle MRI. Muscle biopsy further demonstrated characteristic centronuclear myopathy features.

The heterozygous *MTM1* insertion variant c.1266_1267insATGGTGATAAAAACCATTCA, p.(Gly423MetfsTer48) identified in our proband represents, to our knowledge, the first report of this specific mutation. This variant creates a frameshift leading to a premature termination codon, which is predicted to result in a truncated, non‐functional protein or nonsense‐mediated mRNA decay, consistent with a loss‐of‐function mechanism typical for XLMTM. Segregation analysis confirmed the variant in the proband but not in her parents' peripheral blood, strongly supporting it as a de novo event. The identification of this novel variant expands the mutational spectrum of *MTM1* and underscores that de novo mutations contribute to the presentation of XLMTM in females without a family history.

Although *MTM1*‐related XLMTM is classically described in males, symptomatic heterozygous females often present with a recognizable pattern that can guide diagnosis (Biancalana et al. 2017; Savarese et al. 2016). A large clinicopathological series of symptomatic female carriers highlighted asymmetric weakness and facial weakness, ptosis or ophthalmoplegia as recurrent diagnostic clues, sometimes independent of an affected male in the family (Biancalana et al. 2017). Our patient mirrors these clues—asymmetric proximal and axial weakness with incomplete eyelid closure and mild ocular motility limitations. This phenotypic overlap reinforces *MTM1* as a priority gene for genetic testing in women presenting with a limb‐girdle or axial myopathy phenotype. We further strengthened the comparison between our patient's asymmetric clinical weakness and diffuse yet asymmetric muscle imaging abnormalities against published XLMTM carrier cohorts and case reports. Multiple large‐scale series have established that asymmetric muscle involvement is a hallmark feature of heterozygous MTM1 mutation carriers, even when muscle MRI/CT demonstrates bilateral, widespread myopathic changes (Biancalana et al. 2017; Franken et al. 2022). Takeuchi et al. (2024) reported a female manifesting carrier with bilateral diffuse asymmetric muscle atrophy on CT, who only presented clinically prominent unilateral limb and facial weakness, mirroring the discrepancy observed in our case. This dissociation between diffuse radiological involvement and asymmetric clinical severity arises from tissue‐specific skewed X‐chromosome inactivation: the proportion of cells expressing mutant MTM1 varies independently across distinct muscle groups, resulting in uneven functional impairment despite widespread underlying myodegeneration visible on imaging (Drouet et al. 2008; Biancalana et al. 2017). Unlike symmetric generalized myopathies, the patchy distribution of mutant‐expressing myocytes in female XLMTM carriers generates the characteristic combination of bilateral diffuse radiological signal changes and clinically dominant unilateral weakness, which improves the diagnostic specificity of this disorder among adult limb‐girdle myopathies. Notably, rightward tongue deviation observed in our patient represents a rarely reported phenotypic feature of this disorder. This tongue deviation is most likely attributable to asymmetric lingual muscle weakness secondary to the underlying myopathy. Another notable feature of our patient is generalized areflexia and right‐sided sensorimotor axonal neuropathy without sensory complaints. Routine laboratory testing excluded common alternative causes of peripheral neuropathy, and these findings align with prior published observations (Franken et al. 2022; Takeuchi et al. 2024). MTM1 is ubiquitously expressed not only in muscle but also in peripheral nerves and spinal neurons, and pathogenic MTM1 variants disrupt phosphoinositide metabolism and axonal transport to trigger primary axonal degeneration, while skewed X‐inactivation in heterozygous females further aggravates asymmetric neural and muscular lesions, indicating subclinical peripheral nerve damage can exist without subjective numbness and routine nerve conduction tests should be arranged for symptomatic female carriers (Takeuchi et al. 2024).

Respiratory involvement is a major determinant of morbidity in *MTM1*‐related disease, and authoritative resources note that affected adult females may develop progressive respiratory insufficiency (Biancalana et al. 2017; Savarese et al. 2016). In our case, FVC 53.12% predicted documents moderate‐to‐severe restriction, making respiratory dysfunction a central disease feature rather than a late complication. This aligns with reports of female carriers with prominent restrictive ventilatory defects and underscores that respiratory decline may be under‐recognized in slowly progressive phenotypes (Biancalana et al. 2017; Savarese et al. 2016). Consistent with CHEST 2023 guidance for neuromuscular weakness (Khan et al. 2023), these findings support routine serial pulmonary function testing and timely consideration of noninvasive ventilation when clinically indicated.

The segregation pattern variant present in the proband but absent in parental peripheral blood by Sanger is compatible with a de novo event, yet parental germline mosaicism or low‐level mosaicism cannot be excluded and is directly relevant to recurrence‐risk counseling (Hedberg‐Oldfors et al. 2017; Vincent et al. 1998). Authoritative reviews also acknowledge sporadic presentations in *MTM1*‐related disease and emphasize careful genetic counseling when parents test negative in blood (Lawlor and Dowling 2021). Recent work using genome‐wide phasing and deep sequencing demonstrates that parental mosaicism can be missed by standard testing and can meaningfully alter recurrence‐risk estimates (Lecoquierre et al. 2025), supporting discussion of enhanced testing strategies when reproductive decision‐making depends on risk precision. In this context, our case highlights the practical need to explicitly counsel families about residual recurrence risk and consider higher‐sensitivity approaches per local protocols when appropriate.

### 4.1 Limits

However, there are still some limits in this case. First, respiratory assessment relied on a single pulmonary function test (PFT) report without longitudinal measurements to define trajectory or noninvasive ventilation (NIV) response. Second, segregation was based on peripheral blood only, so low‐level parental germline/somatic mosaicism or tissue‐specific mosaicism cannot be excluded without high‐depth and/or multi‐tissue testing. Finally, standardized quantitative outcomes and unified MRI scoring were limited, and possible modifying factors cannot be fully separated.

## 5 Conclusion

In summary, we report a symptomatic female with a novel, de novo *MTM1* frameshift variant c.1266_1267insATGGTGATAAAAACCATTCA, p.(Gly423MetfsTer48), which expands the known mutational spectrum of XLMTM. This case reinforces that *MTM1*‐related myopathy can manifest in females with a recognizable phenotype characterized by asymmetric axial‐proximal weakness, craniofacial and ocular involvement, and significant restrictive ventilatory impairment, despite normal serum CK levels.

## Author Contributions

**Lijun Chen:** conceptualization, investigation, data curation, writing – original draft. **Yingxiao Bao:** investigation, formal analysis. **Gonglu Liu:** supervision, writing – review and editing.

## Funding

This work was supported by Natural Science Foundation of Zhejiang Province (LY19H090023), and the Key Research and Development Program of Zhejiang Province (2025C02163).

## Ethics Statement

This study was approved by the Ethics Committee of the Second Affiliated Hospital of Zhejiang. University School of Medicine (approval number: IRB‐2017‐022). Written informed consent to: participate was obtained from the patient and participating family members.

## Consent

Written informed consent for publication of this case report and any accompanying images was obtained from the patient. Identifying details have been removed.

## Conflicts of Interest

The authors declare no conflicts of interest.

## Data Availability

The data that support the findings of this study are available on request from the corresponding author. The data are not publicly available due to privacy or ethical restrictions.
